# Effectiveness of peer-led intervention in control of non-communicable diseases in rural areas of Khordha district: study protocol for a cluster randomized controlled trial

**DOI:** 10.1186/s13063-023-07824-w

**Published:** 2024-01-03

**Authors:** Priyamadhaba Behera, Binod Kumar Patro, Arvind Kumar Singh, Susmita Dora, Debapriya Bandhopadhyay, Gautom Kumar Saharia, Anupam Dey, Surama Manjari Behera, Sonu H. Subba

**Affiliations:** 1 Department Of Community Medicine and Family Medicine, All India Institute of Medical Sciences, Bhubaneswar, India; 2 Department Of Community Medicine, Institute of Medical Sciences & SUM Hospital, Bhubaneswar, India; 3 Department of Biochemistry, All India Institute of Medical Sciences, Bhubaneswar, India; 4 Department of General Medicine, All India Institute of Medical Sciences, Bhubaneswar, India

**Keywords:** Non-communicable diseases, Community health, Peer-lead intervention, Diabetes, Hypertension, Dyslipidaemia

## Abstract

**Background:**

The main contributors to death and disability from chronic illnesses in developing nations are elevated blood pressure (hypertension), blood sugar (diabetes mellitus), and blood cholesterol (dyslipidaemia). Even though there are affordable treatments, the treatment gap for these conditions is still significant. Few pilot studies from industrialized nations discuss the value of peer-led interventions for achieving community-level management of blood pressure and blood sugar. This study aims to evaluate the effectiveness of peer-led intervention compared to standard care in achieving control of selected non-communicable diseases (NCDs) in Indian context at 1 year of intervention among people of 30–60 years with hypertension and/or diabetes mellitus and/or dyslipidaemia.

**Methods:**

A cluster-randomized controlled trial will be conducted in villages of two rural blocks of the Khordha district of Odisha from August 2023 to December 2024. A total of 720 eligible participants (360 in the intervention group and 360 in the control group) will be recruited and randomized into two study arms. The participants in the intervention arm will receive a peer-led intervention model for 6 months in addition to standard care. The sessions will be based on the six domains of NCDs — self-care, follow-up care, medication, physical activity, diet, limiting substance use, mental health and co-morbidities. The mean reduction in blood pressure, HbA1C, and blood cholesterol in the intervention arm compared to the standard care arm will be the main outcome.

**Discussion:**

The increasing burden of NCDs demands for newer strategies for management. Peer-led interventions have proven to be useful at the international level. Incorporating it in India will have remarkable results in controlling NCDs.

**Trial registration:**

Clinical Trial Registry of India (CTRI) CTRI/2023/02/050022. Registered on 23 February 2023.

**Supplementary Information:**

The online version contains supplementary material available at 10.1186/s13063-023-07824-w.

## Background and rationale

Non-communicable diseases (NCD) account for 71% of worldwide deaths [1]. Evidence suggests that 85% of premature deaths between 30 and 69 years are estimated to occur in low- and middle-income countries [1]. The cardiovascular mortality rate in India is the highest globally, twice that of the USA and three to five times more than European countries [2]. Currently, India is considered the capital of non-communicable diseases because of its burden. In India, 62% of all deaths and 55% of all disability-adjusted life years (DALYs) can be attributed to NCDs [3].

Hypertension, diabetes mellitus and dyslipidaemia are the most common non-communicable diseases [1]. An estimated 46% of adults with hypertension are unaware that they have the condition [4]. However, only one in five adults (21%) with hypertension have it under control [4]. Similarly, only 23.4% of patients with type 2 diabetes mellitus in India have reasonable glycemic control [5]. ICMR-INDIA study estimated that 79% of men and women had abnormalities in at least one of the lipid parameters [6]. Hypertension, diabetes mellitus and dyslipidaemia share common risk factors. Experiences and theories from India further suggest that a community-based approach, including individual empowerment, community empowerment, and raising critical consciousness, plays a pivotal role in controlling and preventing NCDs [7]. Adherence to pharmacological interventions and lifestyle modifications requires behaviour change that is challenging to achieve at the individual level or clinical care. However, interpersonal influences (such as peers) and environment are vital in changing behaviour [8]. Therefore, peer-led intervention with a community-based approach emerges as a relevant tool for controlling NCDs. Peer-led interventions are not explored in the Indian context for NCDs however found to be successful for alcoholic dependence [9] and weight reduction [10]. Studies from developed countries have shown that peer-led interventions effectively develop NCD knowledge, improve skills, and build confidence in managing their conditions [11]. These studies are primarily focused on glycemic control and improvement of knowledge, and two studies are on reducing blood pressure by peer-led intervention [12–16]. The hour needs to develop and validate integrated, comprehensive peer-led intervention addressing major NCDs that is feasible and culturally adaptable. In this study, along with the development and validation of the peer-led intervention model, we are trying to test the effectiveness peer-led intervention model through cluster-randomized control.

Raised blood pressure (hypertension), blood sugar (diabetes mellitus), and blood cholesterol (dyslipidaemia) are the leading contributing causes of mortality and disability from chronic diseases in developing countries. The treatment gap for hypertension, diabetes and dyslipidaemia remains high even though cost-effective treatment is available. Similarly, compliance with lifestyle modifications (physical activity, dietary recommendations and substance use) remains a challenge at the individual level. To control chronic disease, adherence to lifestyle modifications and pharmacotherapy plays a critical role in changing behaviours. Social norms theory states that the efforts that target interpersonal influences (such as peers) and environment yield better results than those targeted toward the individual in adopting new behaviours or changes in behaviours [8]. Peer supporters generally offer three types of support: emotional, appraisal and informational. Emotional support enhances morale through caring, empathy, encouragement and reassurance. Appraisal support includes:

- Encouraging persistence and optimism for resolving problems.
- Affirming a peer’s feelings and behaviours.
- Reassuring that problems can be solved.

Informational support involves providing advice, suggestions, alternative ideas, feedback, and factual information relevant to the peer’s issue [17]. Therefore, peer-led interventions become relevant to improve self-care, enhance community engagement, support the treatment of NCDs and protect vulnerable populations from a complication of NCDs.

Peer-led interventions to control NCDs are not explored in the Indian context. Few pilot studies are there from developed countries and their importance for controlling blood pressure or blood sugar at the community level. Our study aims to evaluate a peer-led intervention model that is flexible, culturally adaptable and helps to control major NCDs through community engagement improving adherence to lifestyle modifications and pharmacotherapy.

### Objectives

The trial aims to evaluate the effectiveness of peer-led intervention compared to standard care in achieving control of selected NCDs at 1 year of intervention among people of 30–60 years with hypertension and/or diabetes mellitus and/or dyslipidaemia and to determine enablers and barriers to implementation of a peer-led intervention model among people of 30–60 years with hypertension and/or diabetes mellitus and/or dyslipidaemia.

### Trial design

This is a cluster randomized controlled parallel-group trial with a superiority framework. Village is a cluster in the study. Villages will be randomized to two study arms with a 1:1 allocation ratio. The participants in the intervention arm will receive a peer-led intervention model for six months in addition to standard care.

## Methods: participants, intervention, outcome

### Study setting

The trial will be conducted in Mendhasal and Tangi block of Khurdha district of Odisha, India.

### Eligibility criteria

The case definitions and control criteria for hypertension, diabetes mellitus, and dyslipidaemia will be taken from standard national guidelines [18, 19]. Persons in the 30–60 years group with hypertension and/or diabetes mellitus and/or Dyslipidaemia will be recruited in the study based on the following criteria:

#### Inclusion criteria

Cluster level: villages under rural field practice areas of All India Institute of Medical Sciences, Bhubaneswar.

Individual level: persons with hypertension and/or diabetes mellitus and/or dyslipidaemia aged 30–60 years and residing in the same village for the last six months.

#### Exclusion criteria

Cluster level: Villages within 5 km of the community health centre (CHC).

Individual level:

1. Patients who cannot communicate (hearing loss, diagnosed psychosis, etc.)
2. Patient with diagnosed complications such as myocardial infarction, stroke, heart failure and advanced liver disease.
3. Debilitated patients.

### Sample size

The sample size was calculated using nMaster 2.0 software (https://www.cmc-biostatistics.ac.in/nmaster/) with the formula for cluster study design:

$$n=\frac{{\left({z}_{\frac{\alpha }{2}}+{z}_{1-\beta}\right)}^2\left[{p}_1\left(1-{p}_1\right)+{p}_2\left(1-{p}_2\right)\right]\left[1+\left(m-1\rho \right)\right]}{{\left({p}_1-{p}_2\right)}^2}$$

where *p*1 = proportion of outcome in the experimental group

*p*2 = proportion of outcome in the control group

*α* = level of significance

1-*β* = power

*ρ* = Intra-cluster correlation coefficient

*m* = size of the cluster

Considering the proportion of outcome in the control group as 50%, an alpha of 5%, power of 90%, effect size of 20%, design effect of 2.5 and loss to follow-up as 20%, the estimated sample size was 720 participants (40 clusters with 18 participants in each cluster) (Supplementary file 01).

### Allocation concealment

This will be done using sequentially numbered opaque sealed envelopes**.** This will be done by one of the office staff in the department who is not involved in the planning and conduction of the study.

### Sequence generation

**Fig. 1:**
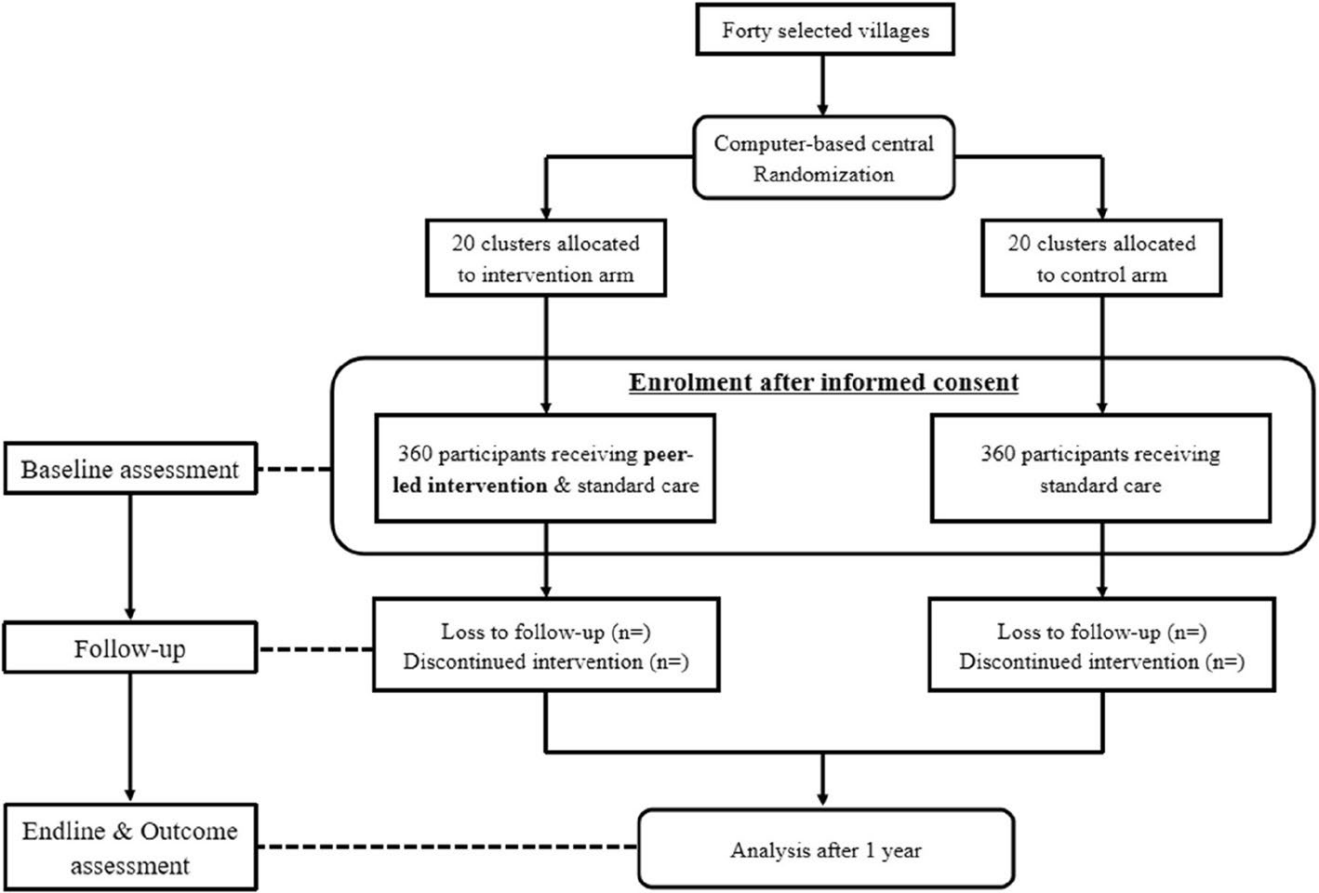
CONSORT flow diagram

Computer-generated random numbers will be used for randomization by the primary investigator (PB). There will be no restriction or stratification. Out of 40 clusters, 20 will receive peer-led intervention and standard care. However, the other 20 clusters will receive standard care alone after randomization. Participants will be enrolled by an investigator (SD). After 1 year, the participants will be assessed in the same manner as the baseline (Fig. 1).

### Intervention

**Fig. 2:**
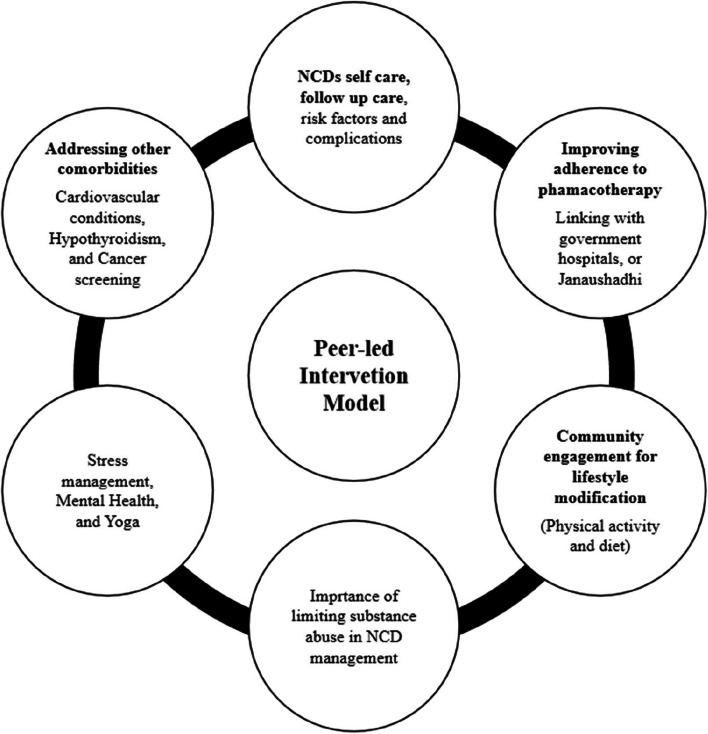
Intervention model

The model will have six sessions (Fig. 2). The intervention will be delivered monthly through trained community health workers supervised by a physician. The sessions will be based on the following six domains: (1) NCDs self-care, follow-up care, risk factors and complications, (2) improving adherence to pharmacotherapy (linking with govt. hospital and/or janaushadhi), (3) community engagement for improving lifestyle modifications (physical activity and diet in NCDs), (4) importance of limiting substance use in NCD management, (5) Stress management, mental health, and yoga, (6) addressing other co-morbidities (cardiovascular conditions, hypothyroidism, cancer screening, etc.). Each peer group in the intervention arm will be provided with a glucometer with glucostrips and a sphygmomanometer. Peer groups will be encouraged to meet fortnightly. The participants will discuss with each other the challenges faced related to domains of intervention and seek appropriate guidance in monthly scheduled peer-led sessions. Through peer education “sharing experiences and learning among people with something in common”, the participants will be empowered to monitor blood pressure and blood sugar and identify feasible interventions for the peer group and individual level. Participants who adhere to lifestyle modifications and pharmacotherapy will be appreciated and share their stories with their peers. At the end of 1 year, intervention participants and the control group will be assessed again. Then the baseline and end line will be compared at individual and cluster levels. The participants in the control arm will receive standard clinical care at baseline [20] and be linked to the nearest govt. hospital for further follow-up care. There will be no special criteria for discontinuing or modifying allocated interventions. We are not storing samples or conducting any genetic or molecular analysis.

*Study tools*: National Non-communicable Disease Monitoring Survey (NNDMS) adult interview questionnaire, Weighing Scale, Stadiometer, Sphygmomanometer, Measuring tape and Glucometer.

**Fig. 3:**
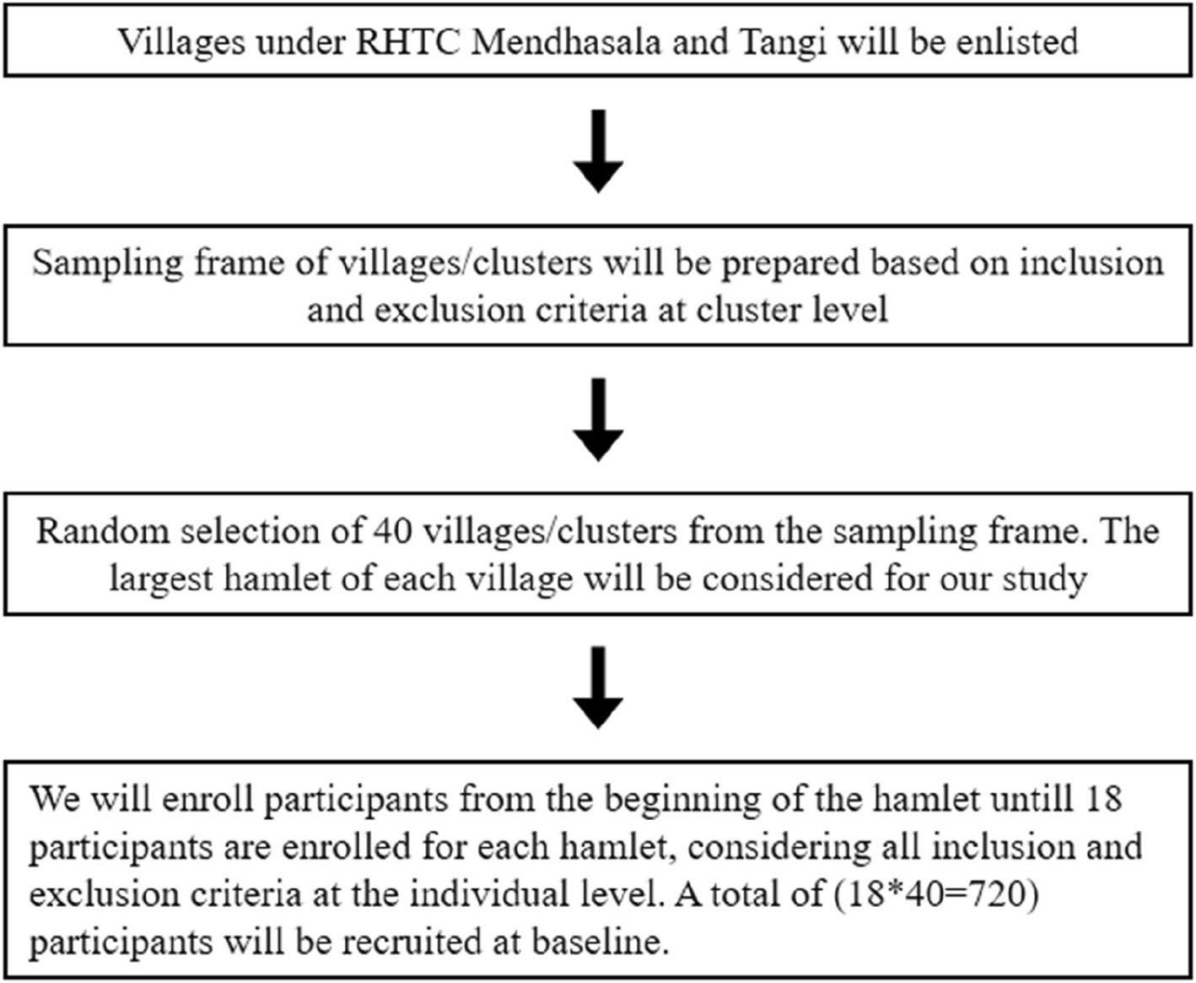
Sampling technique

*Sampling strategy* (Fig. 3)

*Cluster*: Forty villages will be selected randomly from the rural field practice area of AIIMS, Bhubaneswar.

*Participant*: The eligible population will be screened for hypertension, diabetes mellitus, and dyslipidaemia. The process of screening will be continued till 18 participants are recruited from each cluster.

*Baseline assessment*: A total of 720 (40*18) participants will be recruited at baseline after written informed consent. Anthropometry, blood pressure (BP), fasting blood sugar (FBS), post-prandial blood sugar (PPBS), glycosylated haemoglobin (HBA1C), lipid profile, and renal function test (RFT) will be done for all participants, in addition to interviews with NNDMS adult interview questionnaire. Standard guidelines as recommended by govt. of India will be followed for all measurements [21].

### Data collection and management

Data will be collected in epicollect5 and analysed using Statistical Package for Social Sciences (SPSS) Version 25 (IBM Corp.). Department of Community Medicine and Family Medicine, All India Institute of Medical Sciences, Bhubaneswar, had more than 10 years of experience for data collection and data management in various studies which includes national and international collaborative studies and various trials. The standard guidelines will be followed for data entry, coding, and storage. Categorical variables will be presented as percentages or proportions. Continuous variables will be presented as mean and standard deviation. Intention to treat analysis will be done to evaluate the effectiveness of peer-led intervention compared to standard care. The multivariate analysis will be performed to adjust for confounders. For missing variables, the replacement will be tried, if essential, based on the characteristics and quantiles of the variables. There won’t be any interim analysis and no formal trial-stopping rule, as this is a behavioural intervention study with no anticipated problems detrimental to the participant.

The effect of the peer-led intervention will be estimated by Difference in Difference (DID) analysis. A *p*-value of less than 0.05 will be considered statistically significant.

The outcome assessor is not aware of the link with intervention. At the end of one year (12 months) following *outcomes* will be assessed:

1. Improved control status of Non-communicable diseases (hypertension and/or diabetes mellitus and/or dyslipidaemia) in the intervention arm compared to the standard care arm.
2. Mean reduction of blood pressure in the intervention arm compared to the standard care arm.
3. Mean reduction in HbA1C among diabetes mellitus patients in the intervention arm compared to the standard care arm.
4. Mean reduction of blood cholesterol in the intervention arm compared to standard care.

### Adverse event reporting harms and post-trial care

Being a behavioural intervention, adverse events are not expected. Also, there is no anticipated harm and compensation for trial participation. In case of any incidental finding during the study, the participant will be linked to the nearest public health facility or AIIMS, Bhubaneswar.

### Dissemination plans

There will be a minimum of two peer-reviewed publications and two presentations in national or International academic conferences. Lay summary will be prepared and shared with the participants.

## Discussion

According to the social norms’ hypothesis, attempts to adopt new behaviours or alter existing habits that are focused on interpersonal factors (such as peers) and surroundings tend to be more successful than those that are focused on the individual. One of the previous clusters of randomized controlled trials from Kerala, India, has attempted to evaluate lifestyle modifications for lowering the incidence of diseases among those at “high risk” of acquiring T2DM, hypertension, and dyslipidaemia [22]. In India, peer-led treatments have been proven to be effective in treating alcoholism and helping people to lose weight. They have also been used to help people learn about NCDs, improve their abilities, and gain confidence in their ability to manage their diseases [23]. This study aims to evaluate a peer-led intervention paradigm that is adaptable to different cultural contexts, flexible, and aids in the community-based management of major NCDs. If the peer-led intervention model is found to be effective, it will be a potential intervention which can be integrated with the national program of NCDs for larger implications.

## Trial status

Version 2.0, 20/11/2023. Approved by IEC on 12/09/2022. Recruitment of participants will start from 1/12/2023 and will be completed by 30/01/2024.

### Supplementary Information

**Additional file 1.**

**Additional file 2.**

**Additional file 3.**

## Data Availability

All the data related to this study will be available from the Clinical Trial Registry of India within 6 months after the trial is completed.

## Abbreviations

AIIMS: All India Institute of Medical Sciences
CHC: Community health centre
DALY: Disability-adjusted life years
FBS: Fasting blood sugar
HBA1C: Glycosylated haemoglobin
IBM Corp.: International Business Machines Corporation
ICMR: Indian Council for Medical Research
IEC: Institute Ethics Committee
NCDs: Non-communicable diseases
PPBS: Post prandial blood sugar
RFT: Renal function test
SERB: Science and Engineering Research Board
SPSS: Statistical Package for Social Sciences
T2DM: Type 2 diabetes mellitus
USA: United States of America
