# Supplementary material for: Effectiveness of peer-led intervention in control of non-communicable diseases in rural areas of Khordha district: study protocol for a cluster randomized controlled trial
Source: Trials. 2024 Jan 3;25:22. doi: 10.1186/s13063-023-07824-w (PMC10765738; doi:10.1186/s13063-023-07824-w)
Supplement: Supplementary file 1 — Additional file 1. [file 13063_2023_7824_MOESM1_ESM.pdf]

Supplementary File 01

There was no study available for peer-led intervention and NCD control in the Indian context. Based on our experience and expert opinion, we have considered effect size as 20%.

The sample size was calculated using nMaster application (https://www.cmc-biostatistics.ac.in/nmaster/) with the formula for cluster study design-

$$n = \frac{\left(z_{\frac{\alpha}{2}} + z_{1-\beta}\right)^2 [p_1(1-p_1) + p_2(1-p_2)] [1 + (m-1)\rho]}{(p_1 - p_2)^2}$$

Where P1= proportion of outcome in the experimental group

P2= proportion of outcome in the control group

α= level of significance

1-β= power

ρ= Intra-cluster correlation coefficient

m= size of the cluster

Considering the proportion of outcome in the control group as 50%, an alpha of 5%, power of 90%, effect size of 20%, design effect of 2.5 and loss to follow-up as 20%, the estimated sample size was 720 participants (40 clusters with 18 participants in each cluster)

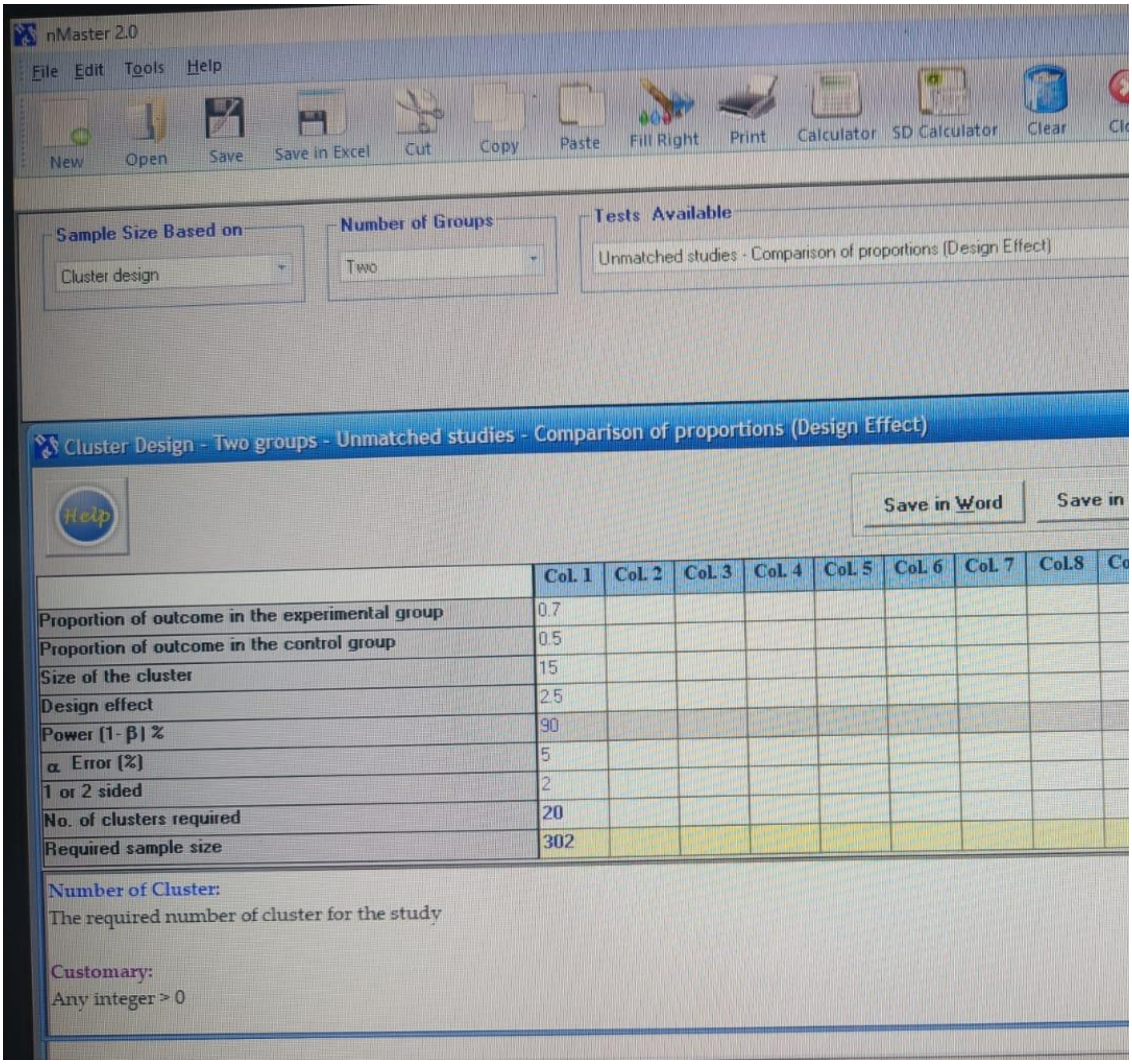

Sample_Size_Peer_led_Intervention_SERB_
