# Supplementary material for: Effectiveness of peer-led intervention in control of non-communicable diseases in rural areas of Khordha district: study protocol for a cluster randomized controlled trial
Source: Trials. 2024 Jan 3;25:22. doi: 10.1186/s13063-023-07824-w (PMC10765738; doi:10.1186/s13063-023-07824-w)
Supplement: Supplementary file 2 — Additional file 2. [file 13063_2023_7824_MOESM2_ESM.pdf]

Participant Information Sheet

DEPARTMENT OF COMMUNITY MEDICINE& FAMILY MEDICINE AIIMS BBSR

Title: " Effectiveness of peer-led intervention in the control of non-communicable diseases in rural areas of Khordha district: A cluster-randomized controlled trial"

I am from the All India Institute of Medical Sciences, Bhubaneswar. I am conducting a study on "**Effectiveness of peer-led intervention in the control of non-communicable diseases in rural areas of Khordha district: A cluster-randomized controlled trial**."

The study is about controlling hypertension and/or diabetes mellitus and/or dyslipidaemia of age 30-60 years for rural people with a peer-led intervention model.

The information obtained from every participant will be kept confidential, and the results will be presented as a summary only. All the data collected will be kept safe, and it will not be communicated to your employer or co-workers under any circumstance. For the disease screening or disease control status, 5 ml blood will be collected at baseline and after 12 months and relevant tests (FBS, HbA1C, Lipid profile, RFT & Fasting Serum Insulin for Diabetes only) will be conducted. The good clinical laboratory practice will be followed during sample collection. The report of these above blood test will be shared with each participant. The information will be pooled and used to derive conclusions that will be shared with the scientific community as a collective only if found relevant. The peer-led intervention model used in the study is found effective; it will behave a larger benefit for people and the community.

There is no risk involved to intervening participants and other village community members. Participants need not spend any money on this study. Your community persons are free to participate or withdraw from this research study at any time. Your decision to participate or withdraw will not affect our relationship. In case of any medical complaints, consultation and referral to a government hospital will be provided.

In case of further information or clarification, you can contact the following at All India Institute of Medical Sciences, Bhubaneswar or the investigator over the telephone.

Dr Priyamadhaba Behera
Assistant Professor
Dept of Community & Family Medicine
AIIMS Bhubaneswar
Mobile: +91 9910830997
