# Supplementary material for: Effectiveness of peer-led intervention in control of non-communicable diseases in rural areas of Khordha district: study protocol for a cluster randomized controlled trial
Source: Trials. 2024 Jan 3;25:22. doi: 10.1186/s13063-023-07824-w (PMC10765738; doi:10.1186/s13063-023-07824-w)
Supplement: Supplementary file 3 — Additional file 3. [file 13063_2023_7824_MOESM3_ESM.pdf]

PARTICIPANT INFORMED CONSENT FORM (PICF)

DEPARTMENT OF COMMUNITY MEDICINE & FAMILY MEDICINE, AIIMS BBSR
Participant ID No. :______________________

Study Title: " Effectiveness of peer-led intervention in the control of non-communicable diseases in rural areas of Khordha district: A cluster-randomized controlled trial"
Investigator: Dr.Priyamadhaba Behera
Phone: +91 9910830997

The contents of attached information sheet dated _________________ that was provided, have been read carefully by me/explained to me in detail, in a language that I comprehend. I have fully understood the contents. I confirm that I have had opportunity to ask questions.
The nature and purpose of study and its potential risks/ benefits and expected duration of study, and relevant details of study have been explained to me in detail. I understand that my participation is voluntary, and that I am free to withdraw at any time without giving any reasons, without my medical care being affected.
I understand that information collected about me from my participation in this research, and sections of any of my medical notes may be looked at by responsible individuals from AIIMS, Bhubaneswar, for these individuals to have access to my records.
I agree to take part in the above study.

Signature/left thumb impression:

Name of the Participant: ___________________________ Date: _________

Father/Mother/Guardian of: ___________________________ Place: ___________

Complete Postal Address: __________________________________

This is to certify that above consent has been obtained in my presence.

Signature of Principal Investigator
Dr. Priyamadhaba Behera

Witness 1 (Name and Signature)

Witness 2 (Name and Signature)
